# Geospatial Analysis of Pediatric EMS Run Density and Endotracheal Intubation

**DOI:** 10.5811/westjem.2016.7.30241

**Published:** 2016-08-22

**Authors:** Matthew Hansen, William Loker, Craig Warden

**Affiliations:** Oregon Health & Science University, Department of Emergency Medicine, Portland, Oregon

## Abstract

**Introduction:**

The association between geographic factors, including transport distance, and pediatric emergency medical services (EMS) run clustering on out-of-hospital pediatric endotracheal intubation is unclear. The objective of this study was to determine if endotracheal intubation procedures are more likely to occur at greater distances from the hospital and near clusters of pediatric calls.

**Methods:**

This was a retrospective observational study including all EMS runs for patients less than 18 years of age from 2008 to 2014 in a geographically large and diverse Oregon county that includes densely populated urban areas near Portland and remote rural areas. We geocoded scene addresses using the automated address locator created in the cloud-based mapping platform ArcGIS, supplemented with manual address geocoding for remaining cases. We then use the Getis-Ord Gi spatial statistic feature in ArcGIS to map statistically significant spatial clusters (hot spots) of pediatric EMS runs throughout the county. We then superimposed all intubation procedures performed during the study period on maps of pediatric EMS-run hot spots, pediatric population density, fire stations, and hospitals. We also performed multivariable logistic regression to determine if distance traveled to the hospital was associated with intubation after controlling for several confounding variables.

**Results:**

We identified a total of 7,797 pediatric EMS runs during the study period and 38 endotracheal intubations. In univariate analysis we found that patients who were intubated were similar to those who were not in gender and whether or not they were transported to a children’s hospital. Intubated patients tended to be transported shorter distances and were older than non-intubated patients. Increased distance from the hospital was associated with reduced odds of intubation after controlling for age, sex, scene location, and trauma system entry status in a multivariate logistic regression. The locations of intubations were superimposed on hot spots of all pediatric EMS runs. This map demonstrates that most of the intubations occurred within areas where pediatric EMS calls were highly clustered. By mapping the intubation procedures and pediatric population density, we found that intubation procedures were not clustered in a similar distribution to the pediatric population in the county.

**Conclusion:**

In this geographically diverse county the location of intubation procedures was similar to the clustering of pediatric EMS calls, and increased distance from the hospital was associated with reduced odds of intubation after controlling for several potential confounding variables.

## INTRODUCTION

Pediatric emergency airway management entails low frequency, high risk procedures for providers in the emergency department and in the emergency medical services (EMS) system. Emergency physicians have the advantage of concentrating these relatively rare procedures among a smaller group of providers compared to many EMS systems. In some EMS systems paramedics intubate a child once every 5–10 years, while in other systems where critical procedures are concentrated among a smaller group of providers, pediatric intubations happen once every 2–3 years for an individual medic.^1^,^2^ Increased exposure to these critical procedures in practice likely enhances skill retention and promotes safety and efficacy.^3^

This study was prompted by the concept that if pediatric endotracheal intubations took place near clusters of pediatric calls, EMS agencies could station specific providers in those areas thus concentrating both general and critical pediatric patient exposure in a smaller group of providers. The primary objective of this study was to determine if endotracheal intubation procedures are more likely to occur in geographic clusters of pediatric calls. We also wanted to know whether pediatric intubations were more likely to happen at greater distances from the hospital potentially due to concern about risks of an unprotected airway during a longer transport. We hypothesized that intubation procedures would occur primarily among locations with high densities of pediatric EMS calls and would be relatively more common with greater transport distance.

## METHODS

### Study Design and Variables

This was a retrospective observational study including all EMS runs for patients less than 18 years of age from 2008 to 2014 in a geographically large and diverse Oregon county that includes densely populated urban areas near Portland and remote rural and wilderness areas. Our primary outcome was the geospatial association between clusters or “hot spots” of pediatric EMS runs and location of endotracheal intubation procedures. The variables collected in the dataset for the study included the following: address of the incident, type of location (home/school), primary impression, procedures performed during the call, transport priority (lights and sirens), ground distance travelled as recorded by the EMS providers, destination hospital, patient age and sex. ArcGIS version 10.2.1 (Redlands, CA) was used for all geographic information systems (GIS) analysis.

### Human Subjects Review

The study was approved by our university’s institutional review board (IRB #00010944), which provided a waiver of informed consent.

### Study Setting/EMS System Characteristics

In this county both public fire and private transport services are dispatched to nearly all 911 calls. The county is served by several fire agencies including large urban and small rural agencies. Transport services are provided by a single private agency throughout most of the county with two small fire districts providing their own ambulance service. Ambulances are staffed with one paramedic and one EMT or Advanced EMT. Fire services have various staffing models based on the agency, but most EMS responses in the urban and suburban parts of the county have a fire crew with at least one paramedic. Paramedics in this county can intubate any age patient. Indications for intubation are broad and include respiratory insufficiency, altered mental state with airway compromise, and situations requiring positive pressure ventilation. Paramedics can use rapid sequence intubation for patients who do not have a gag reflex, using combinations of sedatives (etomidate or midazolam) and paralytics (succinylcholine, rocuronium, or vecuronium) depending on contraindications, medical director preference, and availability of these medications. The protocol for pediatric intubation at the time of the study called for using a bag valve mask (BVM) or a rescue device if two attempts at intubation failed. However, at the time of the study, the King laryngeal tube was the rescue device being used and it was not available in sizes suitable for most children under the age of eight. Providers did have access to oral and nasal airways in all pediatric sizes.

### Geographic Information System (GIS) Analysis

We geocoded incident location addresses using the automated address locator created in the cloud-based mapping platform ArcGIS supplemented with manual address geocoding for remaining cases not coded using the automated method. Using these techniques we successfully matched over 95% of addresses. We then used the Optimized Getis-Ord Gi* spatial analysis feature in ArcGIS to map statistically significant spatial clusters (hot spots) of pediatric EMS runs throughout the county. This tool compares the location of the event of interest in the context of neighboring event locations using an optimized grid that defines the areas of interest. The Gi* statistic calculated for the feature of interest is the z-score and it represents the number of standard deviations from the expected mean number of spatial features per grid box. A relatively high z-score results when the local sum of event locations is higher in one grid box than expected and indicates relatively intense clustering of events or “hot spots.” The tool calculates p-values for the likelihood of a hot spot existing in the grid box. After identifying the hot spots and associated z-scores we superimposed the location of all intubation procedures performed during the study period on the map of statistically significant spatial clusters of pediatric EMS runs.

Next, we displayed population density according to the five-year population estimate from American Community Survey published by the U.S. Census Bureau and displayed the number of residents in each census tract less than 18 years of age with intubation locations.^4^ We displayed the location of fire stations in the county with intubation locations. We obtained fire station locations from the Oregon Metro Regional Land Information System.^5^ Finally, we displayed the location of the intubation locations with the location of hospitals in the area. There are no well-established spatial statistics for comparing the geospatial association between two groups of spatial clusters so we created maps to display all of the data visually to allow readers to make comparisons.

### Non-GIS Analysis

We computed descriptive statistics for all patients in the cohort to compare those who were and were not intubated and identify associations between intubation and transport distance in addition to patient factors. We also conducted a logistic regression to determine if distance traveled from the scene to the hospital was associated with intubation after controlling for several potential confounders. The model for the logistic regression was developed based on *a priori* hypotheses of factors that could be associated with intubation considering the available data.

## RESULTS

We identified a total of 7,797 pediatric EMS runs during the study period and 38 endotracheal intubations. In univariate analysis we found that patients who were intubated were similar to those who were not intubated in gender and whether or not they were transported to a children’s hospital. Intubated patients were transported with lights and sirens more commonly and tended to be transported shorter distances (Table 1). Patients who were intubated were older than non-intubated patients. Table 2 shows the specific types of locations where all pediatric EMS runs and EMS runs where intubations took place.

**Figure 1:**
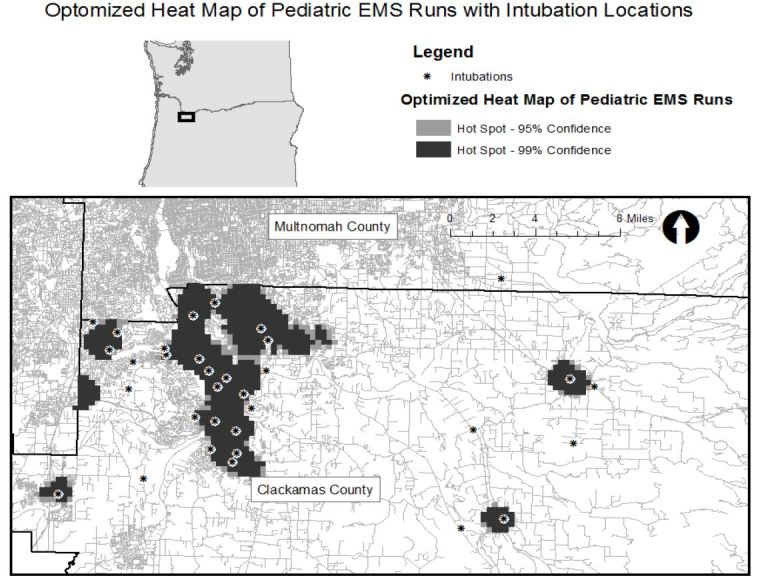
Optimized heat map of pediatric EMS runs with intubation locations *EMS,* emergency medical services.

**Figure 2:**
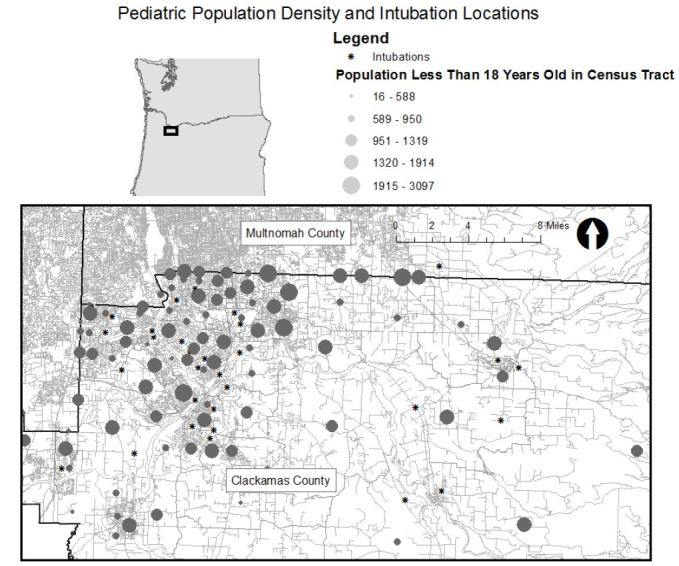
Pediatric population density and intubation locations.

**Figure 3:**
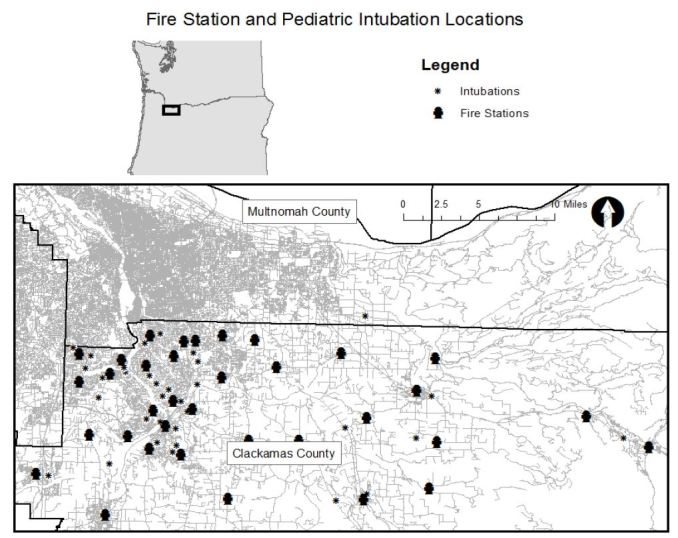
Fire station and pediatric intubation locations.

**Figure 4:**
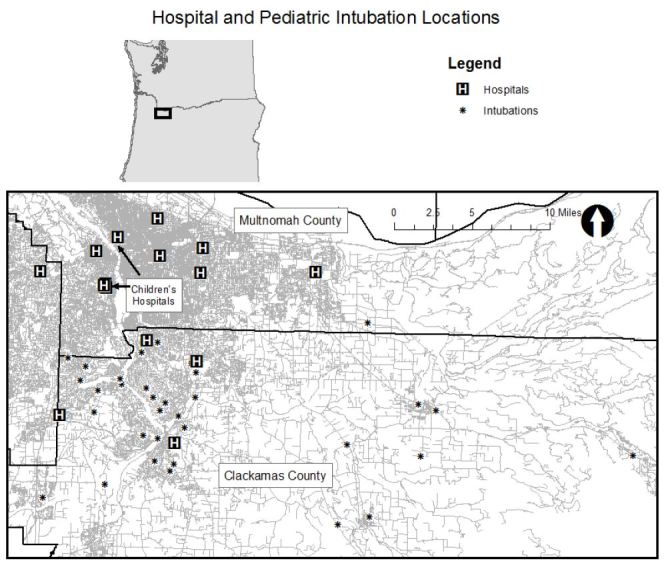
Hospital and pediatric intubation locations.

Figure 1 shows the location of intubations superimposed on hot spot analysis of all pediatric EMS runs with the map extent focused on the portion of the county where hot spots were concentrated. Thirty of the 38 intubations occurred within or immediately adjacent to an area where pediatric calls were statistically significantly clustered. (Map extent excludes four intubations.) Figure 2 displays the pediatric population density by census tract and shows that intubation locations and higher pediatric population density (larger circles) do not appear highly correlated spatially. Finally, Figure 3 shows the relation of fire stations and intubations demonstrating that there is at least one fire station within two miles (straight line distance) of all but one intubation on the map extent. The farthest east intubation, excluded from the displayed map extent, took place approximately five miles from the nearest fire station. Figure 4 displays the location of the acute care hospitals in the area and identifies the location of the two children’s hospitals in the state.

We performed logistic regression analysis with intubation as the dependent variable while controlling for age, miles traveled to the hospital, sex, home as the scene location, and trauma system entry status. We selected this model based on our *a priori* hypotheses of available data that could contribute to likelihood of intubation. We found the older patients, patients who were trauma system entries, and patients who were closer to the hospital tended to be intubated at higher rates. Results of this regression with 95% confidence intervals are displayed in Table 3.

## DISCUSSION

We found that in this geographically diverse county pediatric intubation procedures occur close to spatial clusters of pediatric EMS runs. However, intubation procedures do not seem to cluster near higher densities of children in the general population. Finally, distance traveled to the hospital was not associated with increased odds of endotracheal intubation and in fact there was a trend towards decreased odds of intubation with increased distance.

Pediatric endotracheal intubation is rarely performed by individual providers and thus requires significant resources and training to maintain competency. In addition, there is evidence that pediatric endotracheal intubation may be no better than BVM ventilation, causes harm in some groups, and has high complication rates in the out-of-hospital environment.^6^,^7^ The findings of this study suggest that it may be feasible to adapt airway management practices within an agency to reflect the geospatial distribution of the procedures. An agency could continue to train providers who work in areas where pediatric calls are clustered to intubate children while limiting practice in areas where intubation is unlikely to BVM ventilation or a supraglottic airway. This could promote patient safety while limiting training resources required of the agency. However, this model depends on a workforce that consistently services a particular area and may not apply to settings where fire providers frequently rotate from station to station or ambulances change coverage zones frequently. Further research is needed to validate the findings of this study in other populations and geographic areas.

We found that intubation procedures clustered more closely with pediatric EMS calls rather than the location of larger numbers of children in the community. There are relatively few studies that describe disparities in pediatric EMS care and utilization. A geospatial study identified increased use of EMS among asthmatic children with particularly high EMS utilization among asthmatic children from poor, black, and less-educated populations.^8^ In addition, adults with lower socioeconomic status are more likely to use EMS.^9^ It is possible that intubations cluster near EMS calls rather than the population because EMS may be used by a subset of the population that is more likely to suffer from critical illness and is thus more likely to require intubation. A previous geographic analysis found that pediatric pedestrian injuries are more likely in low income areas.^10^ In addition, socioeconomic factors have been associated with death and healthcare utilization among asthmatic children further supporting the hypothesis that a subset of the pediatric population may be at higher risk for critical illness.^11^,^12^ An alternate hypothesis is that patients in areas of higher socioeconomic status are more likely to be transported by private vehicle, even for a critical illness, and may be under-utilizing EMS resources. This has been suggested in one previous analysis.^13^ Further investigations could explore the potential disparities and differences of EMS utilization in children in various populations and locations.

Finally, we hypothesized that intubation would be more likely to take place at greater distances from the hospital due to the potential perceived risks of an unprotected airway in a long transport. We found that this was not true and in fact there was a trend towards intubation happening closer to the hospital even after controlling for confounders including whether the patient was a trauma system entry. Previous data has suggested that children who require EMS care in rural areas are more likely to suffer from trauma, and are also more likely to have longer transports so it was important to control for this variable.^14^ One possible reason for increased intubations closer to the hospital relates to level of training of staff on scene. A single transport agency covers the vast majority of calls in this county with equal staffing on all vehicles. However, fire department crews operating closer to the hospital are likely to have more professionals on staff and therefore more paramedics relative to more distant areas, which tend to be more rural and often have more volunteers. Relatively more paramedics on scene may make intubation more likely to take place.

We found that older patients were more likely to be intubated compared to younger patients. This may be due to paramedics having increased comfort intubating larger patients who are more adult-like since adults likely comprise the vast majority of airway training and field experience. Post-pubertal patients are generally “adult-like” in anatomy and physiology despite being less than 18 years of age. In some trauma systems patients 15 years and older are treated as adults.

## LIMITATIONS

This study has several limitations to consider. First, this was a retrospective study based on what was documented in the chart by the treating providers. Next, this study was limited to a single EMS agency with specific geography and may not apply to other agencies. EMS agencies who consider adopting scope of pediatric intubation based on geography should conduct their own detailed geospatial analysis.

## CONCLUSION

In this geographically diverse county, it appears that the location of intubation procedures is similar to the clustering of pediatric calls and that distance to the hospital was not associated with increased odds of intubation.

## Figures and Tables

**Table 1:** Patient characteristics in geospatial analysis of pediatric emergency medical service runs and pediatric intubation.

Patient and call characteristic	Non-intubated patients n=7759	Intubated patients n=38	P-value
Mean age (SD)	10.6 (6.1)	13.0 (5.4)	0.014
Sex (% female)	43.6%	47%	0.89
Mean ground distance traveled in miles (SD)	15.9 (13.8)	10.4 (11.3)	0.014
Lights and sirens	8.9%	94.7%	<0.001
Scene is a home	34.0%	42.4%	0.32
Children’s hospital destination	41.1%	52.6%	0.15

**Table 2:** Location characteristics.

	Non-intubated patients n=7759	Intubated patients n=38
Home	2638	14
Medical clinic	410	2
Hospital	909	0
Police station	0	1
Road/street	690	11
Recreation/sports location	630	4
School/government area	534	1
Water (pool/river/lake)	17	1
Missing	977	5
Other	954	0

**Table 3:** Logistic regression analysis.

	Adjusted odds ratio	95% Confidence interval
1 year increase in age	1.081	1.01–1.16
Male sex	0.89	0.45–1.80
1 mile increase in distance to the hospital	0.96	0.93–1.0
Scene location was a home	1.63	0.79–3.35
Trauma system entry	8.26	3.37–20.27
